# 智能响应材料在磷酸化肽和糖肽富集中的应用

**DOI:** 10.3724/SP.J.1123.2022.06026

**Published:** 2022-10-08

**Authors:** Yanqing ZHAO, Wenhui XU, Qiong JIA

**Affiliations:** 吉林大学化学学院, 吉林 长春 130012; College of Chemistry, Jilin University, Changchun 130012, China

**Keywords:** 智能响应材料, 外源响应, 内源响应, 磷酸化, 糖基化, 综述, smart responsive materials, exogenous response, endogenous response, phosphorylation, glycosylation, review

## Abstract

蛋白质的磷酸化和糖基化作为研究最广泛的两种翻译后修饰(PTMs),在疾病的早期无创诊断、预后和治疗评估中表现出越来越大的潜力。蛋白质的异常磷酸化和糖基化经常被用于临床蛋白质组学研究和疾病相关生物标志物的发现。目前已有多种材料被开发用于磷酸化肽和糖肽的富集研究,其中,智能响应材料由于具有独特的响应特性,已被陆续报道用于磷酸化肽和糖肽的富集。智能响应材料可对外界刺激做出响应,发生结构和性质上的变化,将光、电、热、机械等信号转化为生物化学信号。响应分子是决定智能响应材料响应特性的先决条件,它们在不同刺激条件下(如温度、pH、光、机械应力、电磁场等)的可逆异构化将导致材料的宏观物理和化学性质的动态变化。与传统材料相比,智能响应材料可以可逆地“打开”和“关闭”,具有更好的可调控性。由于引起智能材料响应的刺激信号对其性能具有重要的影响,综述根据施加的刺激种类对智能响应材料进行分类,具体分为外源性响应材料和内源性响应材料,且分别总结了外源性响应材料、内源性响应材料以及内外源共同响应材料在磷酸化肽和糖肽富集方面的工作。此外,综述对智能响应材料在磷酸化肽和糖肽富集方面的发展前景进行了展望,并且提出了智能响应材料在其他蛋白质翻译后修饰方面的应用中存在的挑战。

## Abstract (en)

Phosphorylation and glycosylation of proteins, two of the most widely studied post-translational modifications (PTMs), have shown increasing potential in the early non-invasive diagnosis, prognosis, and therapeutic evaluation of diseases. Besides regulating the function of cell membranes and intracellular signal transduction, protein phosphorylation participates in mitochondrial function and cellular and transcriptional metabolism. Protein glycosylation plays an important role in both intracellular and extracellular signal transduction and intracellular endocytosis. Aberrant phosphorylation and glycosylation of proteins are frequently observed in clinical proteomic studies and in the discovery of disease-related biomarkers. There are generally three methods for detecting protein phosphorylation/glycosylation: isotope radiolabeling, western blotting, and mass spectrometry. Mass spectrometry has become the most important and advantageous detection method due to its high throughput and time- and labor-efficiency. However, phosphopeptides and glycopeptides have low stoichiometry and ionization efficiency, and a large number of non-phosphopeptides and -glycopeptides interference. These issues make it difficult to directly detect phosphopeptides and glycopeptides by mass spectrometry. Therefore, the enrichment of phosphopeptides and glycopeptides before mass spectrometry detection is a key step. At present, a variety of materials have been developed for enrichment studies of phosphopeptides and glycopeptides. For example, immobilized metal affinity (IMAC) and metal oxide affinity chromatography (MOAC) methods are mostly used for the enrichment of phosphopeptides. The IMAC mainly uses positively charged metal ions and negatively charged phosphate groups to attract each other for the purpose of enriching phosphopeptides. MOAC materials rely on the chelation of metal atoms and phosphate oxygens to capture phosphopeptides. IMAC and MOAC materials rely on strong interactions between metals and phosphate groups, which often lead to difficult elution. The enrichment method for glycopeptides is mainly based on the difference in hydrophilicity between glycopeptides and non-glycopeptides, which are mainly enriched by hydrophilic interaction chromatography (HILIC). In addition, materials containing compounds such as boronic acid and lectin materials are also widely used for the separation and enrichment of glycopeptides. Smart responsive materials have also been successively reported for the enrichment of phosphopeptides and glycopeptides due to their unique responsiveness and reversibility. Smart responsive materials can respond to external stimuli; undergo structural and property changes; and convert signals such as optical, electrical, thermal, and mechanical into biochemical signals. Responsive molecules are a prerequisite for determining the response properties of smart responsive materials, and their reversible isomerization under different stimuli (such as temperature, pH, light, mechanical stress, and electromagnetic field) will lead to dynamic changes in the physical and chemical properties of materials. Compared with traditional materials, smart responsive materials can be reversibly “turned on” and “off” with better controllability. Exogenous stimuli, including temperature, light, ultrasound, electromagnetic field, and mechanical stress, can be implemented in a specific time and space. Exogenous responsive materials do not depend on changes in the reaction system itself and are non-invasive. Enzymes, pH, redox, solution polarity, and ionic strength are endogenous stimuli. Endogenous responsive materials depend on changes in the reaction system itself, and sometimes the regulation process requires the introduction of other chemicals into the reaction system. The identification, capture, and release of phosphopeptides or glycopeptides can be achieved by modulating the interactions between smart responsive materials and phosphopeptides or glycopeptides (such as hydrogen bonds, and electrostatic and hydrophobic interactions). This review classifies smart responsive materials according to the types of stimuli, which are specifically divided into exogenous and endogenous responsive materials. The enrichment of phosphopeptides and glycopeptides of exogenous/endogenous responsive materials and endogenous/exogenous co-responsive materials are summarized. In addition, we discuss the development prospects of smart responsive materials in the enrichment of phosphopeptides and glycopeptides, and also raised the challenges existing in the application of smart responsive materials in other protein post-translational modifications.

蛋白质的磷酸化和糖基化是蛋白质翻译后修饰(PTMs)中最广泛的共价修饰形式,它们对生物体的生命过程具有重要的调控作用。磷酸化是在蛋白激酶的催化下,将腺嘌呤核苷三磷酸(ATP)或鸟嘌呤-5'-三磷酸(GTP)的*γ*位磷酸基团转移到底物蛋白质的氨基酸残基(常见的是丝氨酸、苏氨酸、酪氨酸)上的过程,而其逆向过程则是由蛋白质磷酸酶去除相应的磷酸基团。磷酸化可调控细胞膜的功能、细胞内信号传导、参与线粒体功能、细胞代谢和转录代谢等^[1⇓⇓-4]^。糖基化是在糖基转移酶的控制下,蛋白质或脂质上的氨基酸残基与糖类形成糖苷键的过程,通常发生于内质网。糖基化在细胞内外的信号传导、细胞间的内吞等过程中都发挥着重要的作用。蛋白质的异常磷酸化和糖基化与一系列病理状况密切相关,例如神经退行性疾病、心血管疾病、肿瘤等^[5⇓⇓-8]^。检测蛋白质磷酸化/糖基化的方法通常有以下3种:同位素放射性标记法、蛋白免疫印迹、质谱分析,其中质谱分析方法由于具有高通量、省时省力等优点,已成为最主要的检测手段^[9⇓⇓-12]^。磷酸化肽和糖肽的低化学计量、低电离效率以及大量的非磷酸化肽、非糖肽的干扰,使得直接采用质谱手段进行检测存在很大的挑战,因此在质谱检测前对磷酸化肽和糖肽的富集成为关键步骤。

已有多种富集策略被用于磷酸化肽和糖肽的富集。对于磷酸化肽的富集策略主要包括固定化金属亲和色谱(IMAC)和金属氧化物亲和色谱(MOAC)等^[13⇓⇓-16]^。IMAC策略主要是利用带正电的金属离子和带负电的磷酸基团相互吸引,达到对磷酸化肽富集的目的^[17⇓-19]^。MOAC策略则是依靠金属原子与磷酸基氧的螯合作用实现对磷酸化肽的捕捉^[20,21]^。IMAC和MOAC策略依靠金属和磷酸基团之间的强相互作用,往往会导致洗脱难的问题,即只有少部分结合的磷酸化肽会被洗脱下来。对于糖肽的富集策略主要是基于糖肽和非糖肽之间亲水性的相互差异,利用亲水相互作用色谱(HILIC)对糖肽进行富集。此外,硼酸亲和法^[22]^、凝集素法^[23]^、肼腙反应法^[24]^等也被广泛用于糖肽的分离富集。

目前,已有多种材料被报道用于磷酸化肽和糖肽的分离富集,如壳聚糖^[25]^、磁性二氧化钛^[26]^、磁性石墨烯^[27]^等。近年来,智能响应材料由于优异的响应性和可逆性在很多领域受到了广泛关注,将其用于磷酸化肽和糖肽分离富集的研究也已陆续被报道。智能响应材料能够以规定的方式对环境或外部刺激做出响应^[28⇓-30]^,可以引起智能材料响应的刺激信号包括光、温度、超声、电磁场、pH、酶、氧化还原等^[31⇓-33]^。根据是否向反应体系内引入化学物质,可将这些刺激分为内源性刺激(pH、酶、氧化还原等)和外源性刺激(光、温度、超声、电磁场等)^[32]^。与传统材料相比,智能响应材料可逆的“打开”和“关闭”特性在化学反应中具有更好的可控性。智能响应材料表现出的这种独特性质是由材料中的响应分子决定的。响应分子在刺激条件下的可逆变化将导致材料宏观性质上的动态变化,如带电性质、亲疏水性等^[32,34⇓⇓-37]^。通过调节智能响应材料与磷酸化肽或糖肽的相互作用(如氢键、静电相互作用、亲疏水相互作用等),可以实现磷酸化肽或糖肽的识别、捕获和释放。

智能响应材料在药物输送^[32,38⇓-40]^、抗癌气体NO的释放治疗^[41,42]^、癌症生物成像^[43]^等的相关综述已有报道。Sun等^[44]^详述了蛋白质PTMs的种类、概念、作用、分离富集的研究现状,进而介绍了智能聚合物(smart polymers)修饰的IMAC、MOAC、分子印迹聚合物等材料在磷酸化肽富集中的应用,以及智能聚合物修饰的硼酸基、酰肼化学、亲水相互作用驱动的两性离子聚合物在糖肽富集中的应用。由于引起智能材料响应的刺激信号对其性能具有重要的影响,因此,本综述首次从刺激种类的角度介绍了智能响应材料在磷酸化肽和糖肽富集中的研究进展。按照刺激信号种类,将智能响应材料分为外源性响应材料和内源性响应材料,总结了外源性响应、内源性响应、内外源共同响应的智能材料在磷酸化肽和糖肽分离富集方面的研究现状以及发展前景。

## 1 外源性响应材料

外源性刺激包括温度、光、超声、电磁场、机械应力等,可在特定的时间和空间下实施^[45⇓-47]^,其中温度和光这两类外源性响应材料应用最为广泛。外源性响应材料不依赖于反应体系本身的变化,具有非侵入性,对生物体友好。另外,良好的时空可调性使得其对反应体系的调控更加简单快速。

### 1.1 温度响应材料

温度响应材料的显著特征之一是拥有临界溶解温度。根据其对温度的不同响应程度,可分为两类:一类是在临界温度以上不发生相变的材料(如*N*-异丙基丙烯酰胺,NIPAm),此时的临界温度为低临界溶解温度(LCST);另一类与上述情况相反,材料在临界温度以下发生相变,例如聚丙烯酰胺(PAAm)、聚丙烯酸(PAA)等,拥有高临界溶解温度(UCST)。其中,NIPAm由于具有优越的温度响应性能,应用最为广泛。NIPAm同时含有亲水基团(酰胺基团)和疏水基团(异丙基基团),温度可以改变这些基团与水之间的氢键作用和疏水作用,从而发生相转变^[40,48⇓-50]^。当温度低于LCST时,亲水酰胺基团与水分子之间的氢键作用大于异丙基与水分子之间的疏水作用,聚(*N*-异丙基丙烯酰胺)(PNIPAm)分子链呈现自由且舒展的无规卷曲构象。反之,当温度高于LCST时,PNIPAm分子链发生脱水聚集,呈现卷曲紧缩的小球构象^[51]^。据此,可将NIPAm单体与磷酸化肽或糖肽的识别单体共聚,得到具有柔性聚合物网络的温度响应型磷酸化肽或糖肽吸附剂^[52]^。

### 1.2 光响应材料

光响应材料由于具有光源波长可调节、可远程操控等优点而备受关注。相对于温度响应材料,光响应材料在实际操作中更为便捷。光响应材料中最为关键的是光敏基团,在光(可见光、紫外光、红外光)的作用下,光敏基团接收光信号,通过光化学反应过程将光信号转化成化学信号^[53⇓⇓⇓-57]^。常见的光敏基团大致包括3类:在紫外-可见光下发生顺反异构的偶氮苯类化合物;通过螺C-O键断裂和闭合,造成分子内电荷变化的螺吡喃类化合物;发生周环化反应的二芳基乙烯类化合物。

#### 1.2.1 偶氮苯类化合物

偶氮苯类化合物(AZB)存在两种光致异构体,在不同的刺激条件下偶氮双键周围发生可逆的反-顺异构化。AZB的反式构型通常是最稳定的,顺式构型可通过紫外光(340~380 nm)照射获得。在可见光照射的条件下,AZB发生从顺势构型向反式构型的异构化^[38,51,58⇓⇓⇓-62]^。对于大多数AZB来说,在室温条件下顺势构型通常是不稳定的,会慢慢向反式构型转变。这种顺反异构的转变不会引起化学键的断裂,但是顺式和反式构型的理化性质有所不同,如分子极性、空间位阻、分子尺寸等。依据这一特性,可以将偶氮苯与糖肽模型相连,赋予糖肽自主装能力以及对光的响应特性,有望将AZB用于糖肽的富集研究^[63]^。

#### 1.2.2 螺吡喃类化合物

螺吡喃类化合物(SP)的吲哚啉和吡喃环部分之间的螺C原子在紫外光照射(或近红外光的双光子激发)下由*sp*^3^杂化向*sp*^2^结构转化,发生开环反应,形成更具共轭性的两性离子部花菁(MC)结构^[64⇓-66]^。SP比较特殊,除了光刺激可以使其异构化外,温度、机械应力以及pH、金属离子等都可以使其发生异构化。在SP的闭环异构体中,两个芳环相互垂直,*π*轨道相互不共轭,因此形成的是无色的闭环结构;MC开环异构体拥有共平面结构,两个芳环产生了大的*π*共轭,从而形成有色结构。MC还可以进一步质子化,得到带有正电荷的MCH^+^。结构上的差异使得两者的化学、物理性质存在明显区别。利用SP异构体之间带电性质以及颜色的区别,可以将其广泛应用于诸多领域,如响应性水凝胶^[67⇓-69]^、光响应太阳眼镜^[70]^、信息防伪^[71,72]^等。由于MC或MCH^+^异构体带有电荷,因此,可以基于静电相互作用对阴阳离子(Cl^-^、F^-^/Na^+^)^[64,73]^或带负电荷的磷酸化肽进行富集^[74]^。

#### 1.2.3 二芳基乙烯类化合物

二芳基乙烯类化合物(DTE)在紫外光的照射下发生周环化反应,由无色具有荧光的开环状态异构为有色无发射的闭环状态;在受到可见光的照射下,闭环状态的异构体又可以重新转化为开环异构体^[75⇓⇓-78]^。这种剧烈的结构和电子特性的改变,导致两种异构体吸收光和发射光能力的截然不同^[79⇓-81]^。一般来说,DTE在开环异构体(紫外区的吸收带,无色)到闭环异构体(可见光区的吸收带,有色)的光环化过程中表现出非常大的光谱位移。这是因为两个稠合噻吩环中的*π*电子离域作用以及取代基电子性质的改变。在DTE的实际应用中有两种思路可供选择:一是利用其本身闭环和开环两种异构体的固有性质,即明显的颜色变化设计光学开关;二是利用两种异构体的荧光特性,通过紫外-可见光刺激设计荧光开关。例如,DTE可以作为非肽生色团修饰在多肽骨架中,进而可以通过紫外/可见光有效控制所得肽模拟物的生物活性^[82]^。

## 2 内源性响应材料

内源性刺激包括pH、氧化还原、酶、溶液极性和离子强度等。其中,pH刺激应用较为广泛,酶刺激的特异性较强。与外源性响应材料不同,内源性响应材料依赖于反应体系本身的变化,并且有时调控过程需要向反应体系引入其他化学物质,具有侵入性,对生物体可能有一定损伤^[46]^。

### 2.1 pH响应材料

pH响应的智能材料一般都含有对酸或碱敏感的物质,如环糊精囊泡、羧酸、硼酸、胺等^[40,83,84]^。随着介质pH值的改变,这些基团发生电离,造成溶液内离子浓度的改变,从而引起材料的形状、亲疏水性等理化性质的改变。pH响应材料的开发研究已被用于肿瘤弱酸(pH 5.8~7.2)微环境中的药物控释^[35,40,85]^。富集糖肽常用的策略—硼酸亲和法也是基于pH响应。在pH调控下,硼酸基团与糖肽上的顺式邻二醇可逆结合,实现糖肽的捕获与释放^[86]^。

### 2.2 酶响应材料

酶响应材料一般由肽及其衍生物组成,这是由于酶的作用底物是由特定的氨基酸序列组成的,研究中使用较多的酶有蛋白酶、磷脂酶、糖苷酶等。酶响应材料的作用机理主要包括催化键的形成和断裂、底物的氧化或还原等^[87⇓-89]^。例如,甘露糖转移酶可与*O*-乙酰氨基葡萄糖(*O*-GlcNAc)共价连接生成酮,生成的酮可与氨基生物素相连,然后通过阳离子交换作用分离富集修饰后的*O*-GlcNAc肽段^[90,91]^。酶响应材料的专一性较强,但是酶的价格昂贵,并且有酶参与的反应通常需要保持合适的环境条件来维持酶的活性,因此在磷酸化肽或糖肽的分离富集方面,此类材料并不是首选。

## 3 智能响应材料在磷酸化肽和糖肽富集中的应用

### 3.1 外源性响应材料

#### 3.1.1 在磷酸化肽富集中的应用

用于磷酸化肽富集的外源性响应材料多为温度响应材料。在诸多用于磷酸化肽富集的温度响应材料中,以NIPAm类材料最为常用。Dai等^[92]^以NIPAm为温度响应分子,异丙烯膦酸(IPPA)作为Ti(Ⅳ)固定配体,两者以可逆加成-断裂链转移聚合(RAFT)反应接枝在二氧化硅微球表面,进而得到单重温度响应的色谱材料silica@p(NIPAm-co-IPPA)-Ti^4+^。通过独特的聚(*N*-异丙基丙烯酰胺)的温度响应特性,捕获的磷酸化肽可以通过改变温度被释放,而无需使用任何可能损坏磷酸化肽的洗脱液。该智能响应材料表现出对磷酸化肽的有效捕获以及快速释放,具有很强的富集潜力。Xia等^[93]^以苯基磷酸作为模板,NIPAm为温度响应分子,制备了具有温度响应特性的分子印迹材料(TMIPs)。当温度为28 ℃时,聚合物的空腔尺寸刚好能够捕获酪氨酸磷酸化肽;而当温度升高至28 ℃以上时,聚合物空腔收缩,从而释放捕获到的磷酸化肽。该温度响应材料既有分子印迹材料对目标生物分子优异的靶向性,同时又具备对温度的响应性,这对开发新的蛋白质PTMs富集材料提供了新的思路。

#### 3.1.2 在糖肽富集中的应用

外源性响应材料对糖肽的富集也多是利用温度响应性质。例如,Moon等^[94]^报告了一种串联的温度响应性多孔聚合物膜反应器(TPPMRs)。作者将温度响应性聚合物聚苯乙烯-马来酸酐-聚异丙基丙烯酰胺(PS-MAn-PNIPAm)涂覆在尼龙薄片上制备温度响应性多孔聚合物膜(TPPM)。在TPPMRs中,一个TPPM用胰蛋白酶固定,对糖蛋白进行水解;另一个则用凝集素固定,用于糖肽富集。PNIPAm在高温下形成胶束腔,改变其形态可以导致酶或凝集素与流经膜的蛋白质或糖肽之间的相互作用增强。进而,作者通过改变温度条件,对TPPMRs富集糖肽的性能进行了评估。在37 ℃时,糖蛋白的水解和糖肽富集都非常有效。将该温度响应材料与纳流液相色谱-电喷雾电离-串联质谱(nLC-ESI-MS/MS)方法联用,可从1.5 μL人血浆样品中鉴定出155种糖蛋白和262种*N*-糖肽。该温度响应材料可以有效地用于糖蛋白的癌症特异性生物标志物的开发。

目前,对磷酸化肽和糖肽富集的外源性响应材料大多是基于NIPAm的温度响应材料。通过改变反应体系的温度,导致材料形貌的改变,进而实现对磷酸化肽或糖肽的可控捕获与释放。而其他类型的外源性响应材料,例如光、电磁场等在磷酸化肽和糖肽富集方面应用较少。另外,温度响应材料虽然不向反应体系中引入其他化学物质,但是这类材料可能会引起局部过热现象,从而导致生物体的损伤。

### 3.2 内源性响应材料

#### 3.2.1 在磷酸化肽富集中的应用

相比于酶响应材料,pH响应材料在磷酸化肽的富集研究中更为常见。例如,Wu等^[95]^制备了一种智能纳米探针,在磁性纳米粒子表面引入聚酰胺树状大分子(PAMAM)接枝的聚(甲基丙烯酸)(PMAA)刷(Fe_3_O_4_@PDA@PMAA@PAMAM)。PAMAM具有丰富且交替有序的氨基,通过调节缓冲液和洗脱液的酸度,可以实现氨基与磷酸基团之间静电和氢键相互作用的可调控性。Fe_3_O_4_@PDA@PMAA@PAMAM对磷酸化肽表现出优异的选择性(*β*-酪蛋白∶牛血清白蛋白=1∶500(质量比))和高灵敏度(1 fmol)。除了上述pH响应材料外,还可以基于超分子化学中金属-配体键的动态性,实现磷酸化肽的可控捕获与释放,进而实现对磷酸化肽的富集。例如,Chen等^[33]^设计了一种二维(2D)金属环芯超分子聚合物,超分子环中包含基于氢键(脲基)和静电相互作用的(Pt基金属环)磷酸识别单元,对磷酸化肽具有强有力的和高度特异性的结合。该智能超分子聚合物材料响应的机理为:当向反应体系中加入四丁基氯化铵(Bu_4_Cl)时,在卤化物的诱导下,2D聚合物分解,释放出捕获的磷酸化肽;当添加三氟甲烷磺酸银(AgOTf)时,2D聚合物重组,用于磷酸化肽的捕获。金属-配体键的动态性质,赋予了智能2D金属环芯超分子聚合物与磷酸化肽之间相互作用的可调控性。

#### 3.2.2 在糖肽富集中的应用

*β*-环糊精是一种典型的天然环状低聚糖,具有疏水空腔和亲水外表面,在纳米材料、分子识别等方面应用广泛。基于主客体作用合成的*β*-环糊精囊泡具有独特的响应特性,在热、紫外光、pH或化学刺激下,可以在球状囊泡和纤维束管状两种结构之间可逆转化。Jia等^[24]^将*β*-环糊精囊泡作为pH响应分子,首次引入到聚合物整体柱材料中,当pH为7.4时,*β*-环糊精囊泡呈现亲水性,可以捕获糖肽;当pH降低至5.0时,*β*-环糊精囊泡转化为纤维管状结构,亲水性降低,从而释放糖肽(见图1)。通过改变溶液的pH,引起pH响应整体柱材料的亲疏水性的显著变化,实现对糖肽的选择性捕获与释放。基于pH响应的糖肽富集材料还有很多种,例如,Qin等^[96]^制备了一种酰肼功能化的pH响应性材料—聚(丙烯酸-co-丙烯酸甲酯)(P(AA-co-MA)),并将其用于糖蛋白/糖肽的富集。当pH变化时,发生可逆的自组装和相分离,即在中性至弱酸性(pH=6.0)溶液中,聚合物在水溶液中溶解性良好;当pH降为2.0时,快速沉淀。这是由于当pH降低时,聚合物与目标糖肽结合,发生大规模自组装过程而产生沉淀。随后,作者使用PNGase F(*N*-糖酰胺酶F)切割聚合物P(AA-co-MA)与*N*-糖肽之间的共价键,实现了糖肽的释放。

**图1:**
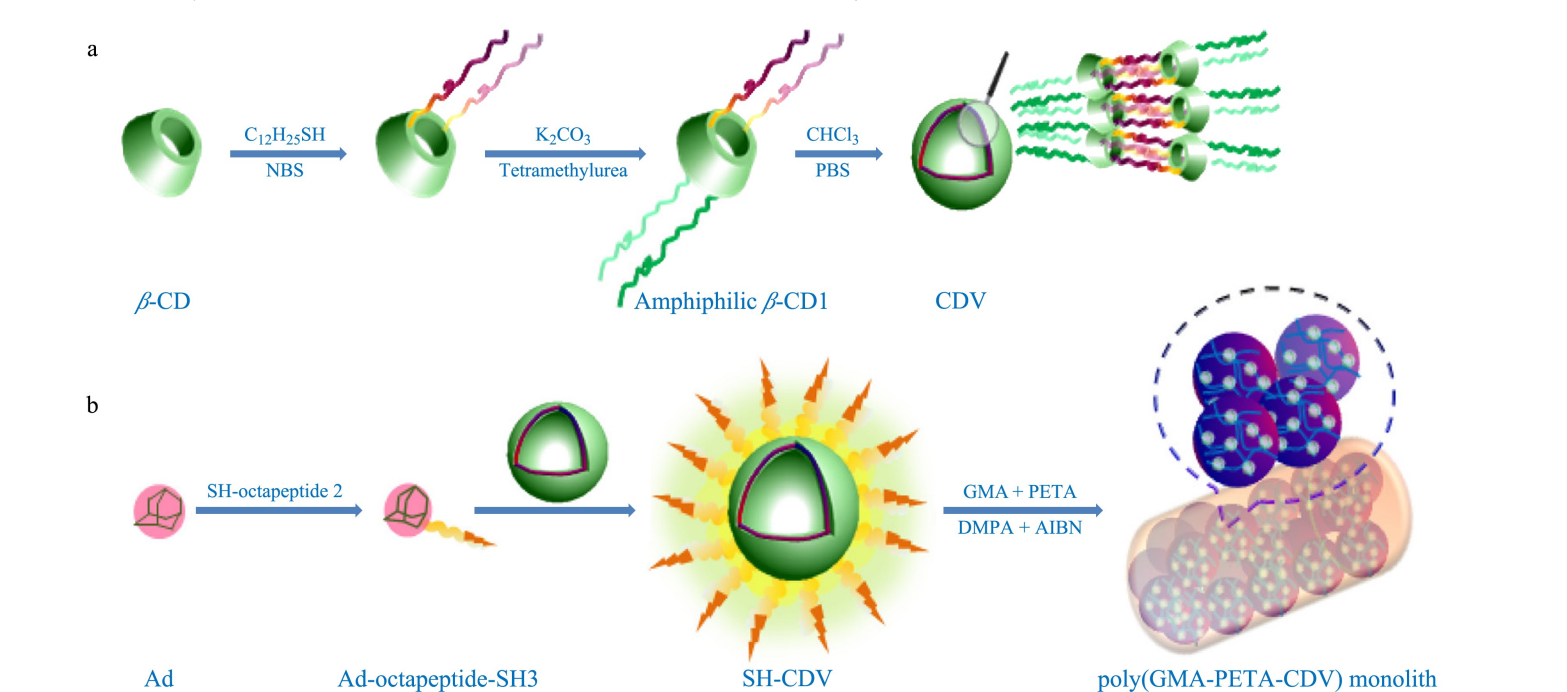
聚(甲基丙烯酸缩水甘油酯-季戊四醇三丙烯酸酯-环糊精囊泡)(p(GMA-PETA-CDV))整体柱材料的合成^[24]^

Liang课题组在响应性材料用于糖肽的富集方面做了大量工作。他们将阿洛糖单元整合到聚丙基丙烯酰(PAM)链中,得到糖类pH响应共聚物材料^[97]^。阿洛糖是一类可对唾液酸糖肽(SGs)表现出特异性结合并且对pH敏感的单糖。阿洛糖对SGs的高特异性主要归因于其与碳水化合物之间的多重氢键相互作用,特异性的糖-糖相互作用成为富集和识别SGs的关键。通过调节溶液的极性和pH,可以智能触发SGs的捕获与释放。随后,该课题组^[98]^制备了乳糖修饰的聚丙烯酰胺智能聚合物(PAM-g-乳糖_0.11_)。乳糖被作为糖肽识别单元,同样基于碳水化合物-碳水化合物相互作用(CCIs),实现了*N*-乙酰神经氨酸(Neu5Ac,一种典型的唾液酸)糖肽的富集。唾液酸分子通过CCIs与接枝乳糖单元的结合诱导聚合物链的构象转变,进一步导致表面形貌、润湿性和刚度的显著和可逆的转换,实现了将微弱的CCIs信号转化为聚合物表面宏观性质(即表面形貌、润湿性和刚度)的转换。PAM-g-乳糖_0.11_凭借对唾液酸的出色识别和响应能力,实现了在复杂样品中SGs的高效富集。

内源性响应材料对磷酸化肽和糖肽的选择性富集研究大多集中于pH响应材料,而酶则通常在磷酸化肽和糖肽富集中用于切割富集完成后材料与肽段连接的共价键。在pH响应过程中,通过调节溶液pH引起材料结构的改变,进而使得材料的理化性质发生显著变化,从而调节了内源性响应材料与磷酸化肽或糖肽之间的相互作用。

### 3.3 内外源共同响应材料

内外源响应材料的制备思路有两种:一是可以将多种响应分子整合,制备内外源共同响应材料;二是对于智能响应材料中的响应分子来讲,有的响应分子可以对多种刺激做出反应,从而得到内外源共同响应的材料。在实际应用中,内外源共同响应材料的设计和制备可以灵敏的感知外界环境的变化并快速地产生动态响应,获得更有效的响应效果。

#### 3.3.1 在磷酸化肽富集中的应用

对磷酸化肽富集来说,由于外源性响应材料大多是基于NIPAm的温度响应材料,因此,内外源共同响应材料通常是将一种内源性响应单体与NIPAm温度响应单体共聚,从而赋予材料多种刺激响应。例如,Sun等^[52]^选取对羧基苯基硫脲(ATBA)与柔性的聚*N*-异丙基丙烯酰胺(PNIPAm)网络共聚,得到三重响应的多磷酸化肽(MPPs)富集材料。PNIPAm具有智能氢键网络,将适当的生物识别单元(ATBA)整合在PNIPAm中,聚合物链的构象发生转变,并影响目标生物分子的捕获与释放。可以通过调节溶剂极性、pH、温度来调节材料的多重氢键作用,从而实现对多磷酸化肽的可逆调控吸附与释放。该智能响应材料对MPPs具有高吸附容量(463 mg/g)和回收率(90%)。此外,作者还从Hela S3细胞裂解物中鉴定出大量多磷酸化位点。这种内外源共同响应材料对MPPs具有高富集选择性,表明其在磷酸化蛋白质组学研究中的巨大潜力。

SP类化合物是一类可对多种内源或外源刺激同时产生响应的响应分子。在不同刺激条件下,电中性的闭环态SP与带电的MC或MCH^+^开环态之间具有可逆的相互异构,这一独特的性质赋予了它广泛的应用。Jia等^[74]^通过简单的酯化反应制备了螺吡喃修饰的磁性纳米粒子(MNP-SP),并将其用于复杂生物环境中磷酸化肽的富集。该项工作同时引入了光和pH双重刺激,在紫外光和酸的刺激下,MNP-SP异构为带正电的MNP-MCH^+^材料,基于静电相互作用,实现了对磷酸化肽的选择性捕获(见图2)。借助MALDI-TOF-MS分析技术,作者对不同刺激条件下材料的富集能力进行了测试。结果表明,与单一内或外源刺激相比,紫外光和pH的内外源共同刺激使得材料的富集能力显著提高。该智能响应材料MNP-SP对磷酸化肽的检测具有高的灵敏度(检出限为0.4 fmol)和良好的可重复使用性(6次循环)。当用于富集脱脂牛奶、人唾液和人血清样品中的磷酸化肽时,该材料被证明是富集复杂生物环境中低丰度磷酸化肽的理想吸附剂。

**图2:**
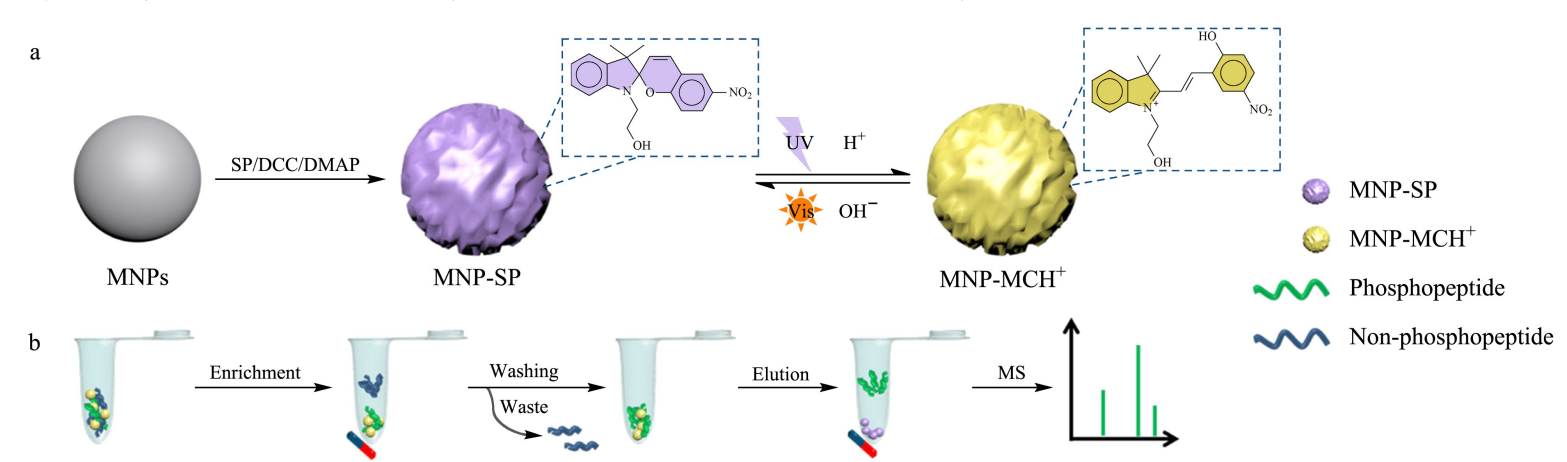
MNP-SP的(a)合成方案和(b)选择性捕获磷酸化肽的过程^[74]^

#### 3.3.2 在糖肽富集中的应用

内外源共同响应材料对于糖肽的富集往往是基于NIPAm温度响应单体与其他pH响应单体的结合。例如,Menzel等^[99]^合成了具有温度响应性和pH响应性的两亲性嵌段共聚物聚(L-谷氨酸)-b-聚(*N*-异丙基丙烯酰胺)(pGA-b-pNIPAM)。聚-L-谷氨酸(pGA)不仅具有亲水性,而且对pH较为敏感,即随着pH值的降低,构象从无规卷曲变为*α*-螺旋结构。作者研究了pGA-b-pNIPAM颗粒的形成,以及pGA糖冠与凝集素的相互作用。结果表明,该双重响应性pGA-b-pNIPAM材料温度在LCST以上时形成了具有疏水核心和糖基化表面的颗粒,并且pGA糖冠与凝集素的这种相互作用使得pGA-b-pNIPAM颗粒有望用于糖蛋白的富集研究。

将NIPAm与糖肽的经典富集策略—硼酸亲和法结合,可以设计基于温度和pH的内外源共同响应材料,并用于糖肽富集。Ye等^[22]^提出了将高密度的硼酸(APBA)配体固定在热响应性嵌段共聚物刷上,得到内外源共同响应材料p(NIPAm-b-pBA),通过固定的硼酸与糖肽的协同多重共价结合,实现了糖肽的选择性富集。将p(NIPAm-b-pBA)修饰在二氧化硅微球表面,得到聚合物杂化材料Si@pNIPAm-b-pBA。该纳米复合材料对糖肽的结合能力可通过控制pH和温度来调节,为开发可同时用于生物分离和生物医学的复合材料提供了新的机遇。

到目前为止,内外源共同响应材料在设计思路上大多是基于温度和pH响应,而关于其他内外源响应材料的研究较少。内外源共同响应材料具有多样化响应特性,可以更好地适应复杂生物环境的变化。相比于单一内或外源响应材料,内外源共同响应材料有望获得更好的响应效果。

## 4 结论与展望

智能响应材料具有卓越的响应性、可逆的构象转换以及优异的可调控性等显著优势,在不同的内/外源刺激条件下,材料的各种宏观理化性质会发生显著变化,这是常规材料所不能实现的。将智能响应材料应用于磷酸化肽和糖肽的富集,主要是基于在该类材料中设计可以精确识别磷酸化肽和糖肽的识别单元。例如,对于磷酸化肽来讲,可以寻找基于氢键(硫脲基、酰胺、胺类等)或携带正电荷基团的识别单元;糖肽则可以选择亲疏水性质有显著变化的识别单元。但是,已研究的富集磷酸化肽或糖肽的智能响应材料大多是基于温度和pH响应,而对其他响应类型研究较少,未来期待开发更多响应类型的富集磷酸化肽或糖肽的智能响应材料。另外,针对磷酸化肽和糖肽富集的智能响应材料设计思路可以扩展到其他的蛋白质PTMs,如乙酰化、泛素化和甲基化等。但是,目前对于其他PTMs肽或蛋白识别单元的设计还存在着较大的挑战。相信随着科技的发展,在未来的工作中智能响应材料可以更加广泛地应用于多种PTMs的分离富集研究。
